# Improving Diagnosis and Management of Opioid-Induced Constipation (OIC) in Clinical Practice: An Italian Expert Opinion

**DOI:** 10.3390/jcm13226689

**Published:** 2024-11-07

**Authors:** Giustino Varrassi, Giuseppe Casale, Maria Grazia De Marinis, Francesco Dentali, Paolo Evangelista, Gino Gobber, Gaetano Lanzetta, Pierangelo Lora Aprile, Maria Caterina Pace, Piero Portincasa, Franco Radaelli, Andrea Ungar

**Affiliations:** 1 Fondazione Paolo Procacci, 00193 Rome, Italy; 2 Fondazione Antea, 00135 Rome, Italy; 3 Fondazione Policlinico Campus Bio-Medico, Università Campus Bio-Medico di Roma, 00128 Rome, Italy; 4 Dipartimento di Area Medica, Asst Sette Laghi, SC Medicina Generale, Università dell’Insubria, 21100 Varese, Italy; 5 School of Medicine, L’Aquila University, 67100 L’Aquila, Italy; 6 Italian Palliative Care Society, 38100 Trento, Italy; 7 INI UniCamillus, Saint Camillus International University of Health and Medical Sciences, 00046 Rome, Italy; 8 Italian College of General Practitioners and Primary Care, 50100 Firenze, Italy; 9 Department of Anesthesia, Luigi Vanvitelli University, 80133 Napoli, Italy; 10 Division of Internal Medicine “A. Murri”, Department of Precision and Regenerative Medicine and Ionian Area (DiMePre-J), University of Bari “Aldo Moro”, 70121 Bari, Italy; 11 Internal Medicine, Valduce Hospital, 22100 Como, Italy; 12 University of Florence and Azienda Ospedaliero-Universitaria Careggi, 50100 Firenze, Italy

**Keywords:** opioid, opioid-induced constipation, diagnosis, management, PAMORAs, clinical practice, primary care

## Abstract

Opioid-induced constipation (OIC) is a very common and troublesome gastrointestinal side effect following the use of opioids. Despite existing international guidelines, OIC is largely underdiagnosed and undertreated. ECHO OIC is a European project designed to improve the diagnosis and management of OIC at the primary care level. The next phase of the ECHO OIC project is to review and adapt the proposed European pathway at national level, considering the local patient journey and clinical practice. A multidisciplinary group of 12 Italian experts reviewed and discussed the European path and formulated a seven-step guide for the practical management of OIC that is also easily applicable in primary care: 1. When prescribing long-term opioids, the physician should inform the patient of the possibility of the onset of OIC; 2. At opioid prescription, doctors should also prescribe a treatment for constipation, preferably macrogol or stimulant laxatives; 3. The patient should be evaluated for OIC within the second week of initiating opioid treatment, by clinical history and Rome IV criteria; 4. In the presence of constipation despite laxatives, prescription of a PAMORA (Peripherally Acting Mu Opioid Receptor Antagonist) should be considered; 5. When prescribing a PAMORA, prescribing information should be carefully reviewed, and patients should be accurately instructed for appropriate use; 6. Efficacy and tolerability of the PAMORA should be monitored regularly by Bowel Function Index, considering a cut-off of 30 for the possible step-up of OIC treatment; 7. After 4 weeks of treatment, if the efficacy of PAMORA is deemed inadequate, discontinuation of the PAMORA, addition of an anti-constipation drugs, change of opioid type, or referral to a specialist should be considered. Spreading knowledge about the OIC problem as much as possible to the health community is crucial to obtain not only an early treatment of the condition but also to promote its prevention.

## 1. Introduction

Opioids are a class of well-known analgesics, whose use has grown in recent years. In the United States (US), about 4–5% of the population are prescribed opioids, mainly for noncancer pain, although the proportion of consumers has grown a lot in recent years [1,2]. In the European Union (EU), the consumption of opioid analgesics has sharply increased from the late 1990s to the mid-2010s by almost 40%, more in Western/Northern countries than in Southern/Eastern ones [3]. Although remaining much lower than in the US and the EU, opioid use is also growing steadily in Italy [3,4]. Opioids are associated with several side effects which include sedation, physical dependence, respiratory depression, and gastrointestinal (GI) side effects. Opioid-induced constipation (OIC) is the most common GI effect of opioids, affecting 40–80% of patients on opioids [5,6,7], and with a significant impact on their quality of life (QoL) [8]. OIC is primarily mediated through the agonist action of opioids on the enteric µ-receptors, which causes increased non-propulsive contractions, inhibition of water and electrolyte excretion, and increased colonic fluid absorption [9,10]. The consequences are delayed GI transit, hardening of stool, and constipation. Opioids also increase anal sphincter tone impairing the defecation reflex [11].

Unlike many of the other side effects of opioids (respiratory depression, nausea, sedation), constipation generally does not improve over time, and therefore should be anticipated, monitored, and addressed throughout the opioid treatment course [12]. Conversely, despite its frequency, OIC is often underdiagnosed and undertreated. Awareness of this complication of opioid therapy is low and its impact on patients’ QoL is underestimated by physicians, particularly among non-specialists [13]. A European patient survey revealed that almost a fifth of respondents were dissatisfied with their current constipation treatment and about one-third questioned the balance between the analgesic effects of opioids and constipation side effects [14]. On the other hand, in an Italian survey conducted among opioid-prescribing physicians (N = 501), about half of whom were general practitioners, it was revealed that up to 40% did not regularly check patients’ intestinal function and OIC symptoms and 67% did not feel adequately educated about OIC [15]. Repeating the survey one year later, although showing considerable progress toward standardization of diagnosis and treatment of OIC, confirmed that the need for further educational and training efforts was still present [16].

**Figure 1:**
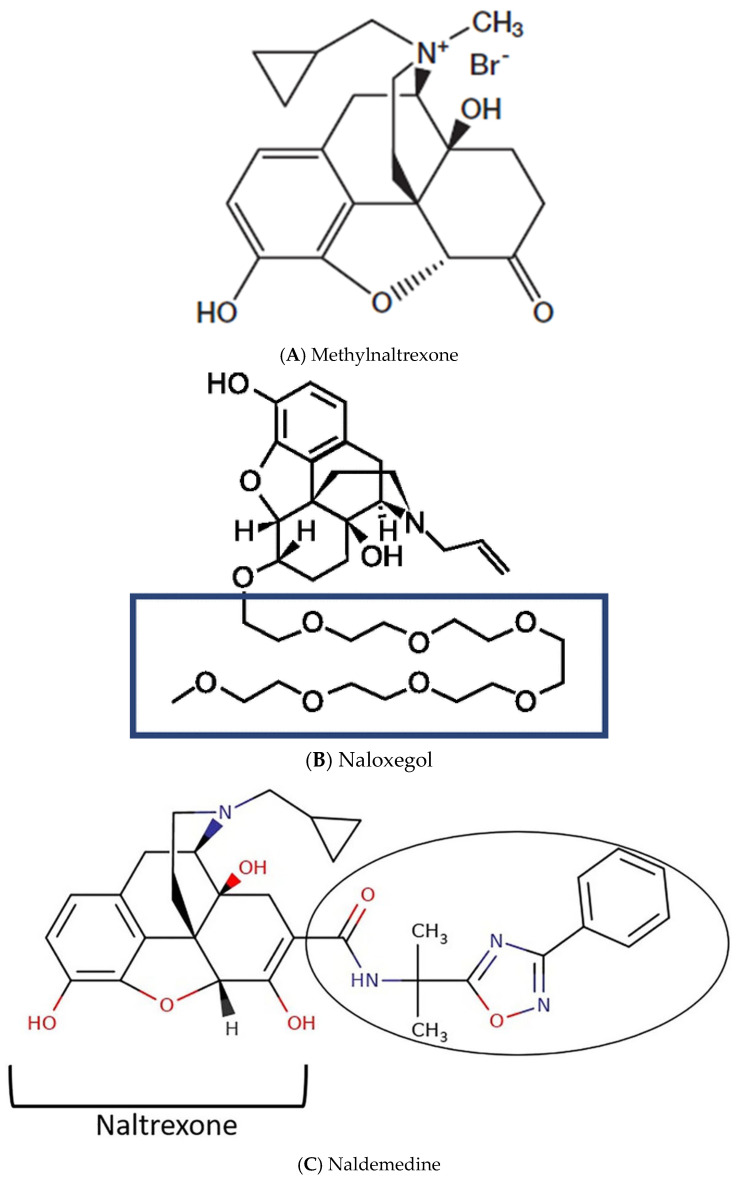
Molecular formulas of PAMORAs approved for opioid-induced constipation.

Prophylactic non-pharmacological and pharmacological approaches are recommended for the initial management of OIC [17]. Non-pharmacological approaches include increasing the intake of fluids and dietary fibres and physical activity, recommendations that are not suitable for many opioid users, such as patients with advanced cancer or debilitated elderly patients with severe chronic pain. Laxatives are the first-line pharmacological treatment for OIC. However, for reasons mostly related to poor prescribing and monitoring they are often ineffective. In recent years, the peripherally acting μ-opioid receptor antagonists (PAMORAs) have been developed, a new class of drugs counteracting the specific action of opioids, which include methylnaltrexone, naloxegol, and naldemedine (Figure 1). Several international guidelines address the diagnosis and treatment of OIC providing recommendations for the management of the condition [17,18,19,20]. However, there is a lack of awareness of these guidelines [21,22] and PAMORAs are used by a minority of opioid-prescribing physicians [15].

The EnhanCing Healthcare Outcomes in Opioid-Induced Constipation (ECHO OIC) project started with the aims of (i) devising strategies for different stakeholders to improve the diagnosis and management of OIC, and (ii) favouring the transfer of care of patients on long-term opioids from secondary to primary care [23]. A multidisciplinary European Steering Committee has been working on the project during the past year to develop recommendations for the careful and conservative use of opioids and improved communication between specialists and non-specialists. A simplified pathway for OIC management for primary care physicians was finally developed and presented at the European Pain Federation 2022 Congress [21]. The proposed pathway focuses on educating the patient about OIC at the time of opioid prescription, properly assessing OIC and its response to laxatives, prescribing PAMORAs in laxative-resistant patients, closely monitoring responses to PAMORAs, and eventually on the opportunity to be referred to a specialist. The next step of the ECHO OIC project was intended to review and adapt the pathway proposed by the European committee at the individual country level, taking into account the local patient journey and clinical practice.

This article summarizes the expert opinion of a multidisciplinary panel of Italian professionals involved in OIC management, which discussed the European output and, consistently with the existing international guidelines, developed a practical OIC diagnosis and management pathway suitable for the Italian clinical practice.

## 2. Methods

The Italian expert panel was composed by 12 members including specialists in pain therapy, oncology palliative care, internal medicine, and gastroenterology, two general practitioners, and a professional nurse. The panel met in Rome on 5 December 2022. The European pathway [21] was reviewed and extensively discussed based on the existing literature and personal expertise and an expert opinion about 7 steps to be considered in patients on chronic opioid therapy to prevent and manage OIC was formulated. The expert opinion was focused on providing practical indications for the effective management of OIC in the secondary and primary care setting.

## 3. Expert Opinion

1. *When prescribing opioids for an expected period of at least 2–3 weeks, the physician should inform the patient of the possibility of the onset of OIC and explain in detail the characteristics of the condition.*

While the culture of pain therapy has become widespread in recent years, awareness of the importance of relieving constipation secondary to opioid therapy has not well known. This means that paradoxically these patients do not suffer so much from the pain, which is adequately controlled by therapy, but rather suffer from the symptoms of pain therapy-induced constipation, which can be quite severe, and impact the QoL. Patients on opioids typically have major medical needs and opioid side effects are often considered a secondary issue compared to their main underlying condition. Awareness about this issue is somewhat higher in the oncology field, where oncologists are used to managing the iatrogenic effects of therapies and patients are more careful about reporting them. On the other hand, patients with chronic noncancer pain account for between 40% and 60% of patients on chronic opioid therapy and are less prone to complain to their physicians about OIC [24]. In the elderly, for example, who represent a large proportion of patients with chronic noncancer pain and suffer from constipation more frequently than younger patients, OIC is largely underreported and underrecognized, being considered a fatal consequence of age and reduced motility [25,26]. Therefore, it is the physicians’ responsibility to make patients aware at opioid prescription that a certain degree of constipation occurs in nearly all patients taking opioids [27]. Patients should also be informed that constipation is not defined exclusively by a reduction in stool frequency, but also by symptoms such as bloating, straining, hard stool consistence, incomplete bowel movements and abdominal discomfort. Nurses play an important role in this information task since they often have a more open and prolonged dialogue with patients. Adequate education of the patients is essential to prevent self-withdrawal or dose reduction of opioids, which occur in 20–30% of patients [28], leading to ineffective pain relief, reduced health-related QoL, and further healthcare costs.

2. *At opioid prescription, doctors should prescribe a treatment for constipation, preferably macrogol or a stepped protocol of a stimulant or osmotic laxative that allows escalation to the highest label-recommended dose if needed. Patients should be informed that if constipation occurs and persists for 3 days despite use of the prescribed laxative, they should contact their physicians for treatment review.*

Changes in diet or lifestyle are often unacceptable by elderly, cancer, or chronic pain patients. Enemas and some manoeuvres aimed at facilitating bowel habits are not only disturbing and painful but can even worsen constipation. Self-prescriptions and imaginative home remedies do not help and can lead to drug interaction problems or complicate the control of opioid side effects. In line with existing guidelines [17,18,19,20], the panel recommends laxatives as first-line treatment and believes that it is worth prescribing them from the very beginning of a prolonged opioid therapy, to try to prevent OIC. Bowel function, including evacuation frequency and stool form (based on a validated assessment tool) [29] are assessed at baseline before starting the opioid therapy for subsequent assessments during opioid therapy. The choice between taking the laxative as prophylaxis or at the very first symptoms of constipation should be discussed between the doctor and the patient on a case-by-case basis.

Laxatives are a broad class of agents with quite different mechanisms of action, including stool softeners, lubricants, osmotic laxatives, and stimulant laxatives. The panel pointed out that not all laxatives are appropriate for OIC. For example, fibre-based bulking agents may worsen abdominal distension. Prokinetics also have very little efficacy. Osmotic laxatives are poorly absorbed by the gut and act as hyperosmolar agents, increasing the water content of stools, which become softer and easier to pass [30]. Polyethylene glycol [PEG]/macrogol is an acceptable first-line agent in OIC, since it has a very favourable safety profile, being inert and reaching the colon intact, where it increases stool mass by hydration. Its effectiveness over placebo is supported by a controlled clinical trial [31]. Stimulant laxatives such as bisacodyl, anthraquinones (e.g., senna), and sodium picosulfate may be another option for first-line OIC management, although their use is not supported by controlled clinical trials but only by a long and extensive clinical practice.

Resistance to laxatives is defined by the Italian Medicine Agency (AIFA) in its Note 90 regarding reimbursement of prescribed PAMORA as a “lack of response after 3 days” [32]. Therefore, experts recommend that patients be made aware that after 3 days of laxative-resistant OIC they should inform their doctor for a possible review of therapy.

3. *The patient should be evaluated for OIC at the latest within the second week of initiating opioid treatment, thorough history and assessment of Rome IV criteria or an appropriate assessment tool.*

The use of laxatives was often shown to be insufficient in preventing/relieving constipation in opioid-taking patients [33]. The close monitoring of the occurrence of OIC should be undertaken after the initiation of opioid therapy. OIC may present immediately when a patient takes the opioid, or it may present gradually during opioid therapy [34]. A first check is suggested within the second week of opioid treatment to assess the presence of OIC according to Rome IV criteria (Table 1) [35,36,37]. The Rome criteria emphasize how straining, and stool consistency are criteria as important as the frequency of evacuations to define constipation, which is not so obvious for “non-specialists”. It is worth underlining the importance of a careful history to investigate other possible concomitant causes of constipation and to evaluate the symptoms and their duration and progression, possibly with the aid of the Bristol Stool Scale, useful to assess subsequent changes in the shape and consistency of the faeces [29]. The Bowel Function Index (BFI) should also be used to assess refractoriness to the first-line therapy. The BFI is a validated scale for the quantitative assessment of bowel function, that was developed precisely to evaluate bowel function in opioid-treated patients with pain [38]. It is a clinician-administered tool that easily and quickly measures OIC from the patient’s perspective and includes three variables: ease of defecation, feeling of incomplete bowel evacuation, and personal judgement of constipation, using a visual analogue scale from 0 (freedom of symptoms) to 100 (maximum difficulty or the most severe symptom) and referring to the previous 7 days. Although not yet validated in the Italian language, the panel believes that the BFI is easily usable in clinical practice and sufficiently sensitive to detect variations in the severity of OIC. The step-up of the OIC therapy should be considered for a value of BFI ≥30 [39]. The Victoria Bowel Performance Scale (revised) is another three-item simple assessment tool that has also been shown to be effective [40].

4. *In the presence of constipation despite a stepped laxative protocol, prescription of a PAMORA (Peripherally Acting Mu Opioid Receptor Antagonist) should be considered.*

Since OIC results from a specific effect of opioids on enteric µ-receptors, targeted therapy should be prescribed after a first attempt with laxatives. Three PAMORAs are currently available for the treatment of OIC: methylnaltrexone, naloxegol, and naldemedine. PAMORAs bind to peripheral opioid receptors, blocking their activation by exogenous opioids within the gastrointestinal tract, thereby preventing or reducing constipation. PAMORAs have specific properties such as low lipid solubility, large structure, and strong polarity, which prevent them from crossing the blood-brain barrier at therapeutic doses so that they do not affect the analgesic activity of opioids [10,41,42,43]. The latest American Gastroenterological Association (AGA) guidelines for OIC management recommend PAMORAs for laxative-refractory OIC, acknowledging naldemedine as the one with the highest quality of evidence [17]. Unlike methylnaltrexone and naloxegol, naldemedine antagonizes all the peripheral opioid receptors—∂-, ƙ- and µ-receptors—variably distributed through the GI tract [44]. The clinical efficacy of naldemedine was assessed in 3 RCTs, i.e., the COMPOSE 1, 2, and 3 trials [45,46]. COMPOSE-1 and 2 showed a significant superiority of naldemedine versus placebo in the percentage of treatment-responders, defined as patients achieving at least 3 spontaneous bowel movements per week (pooled risk ratio 1.51 [95% CI, 1.32 to 1.72]). COMPOSE-3 assessed the long-term effects of naldemedine (52 weeks), showing a significant increase in bowel movement frequency, and improvement of OIC symptoms and patients’ quality of life compared to placebo. Methylnaltrexone and naloxegol are also strongly recommended by the latest AGA guidelines, although the quality of the evidence for these PAMORAs is considered low and moderate, respectively [17]. Methylnaltrexone is supported by several RCTs; however, not all have ≥3 rescue-free bowel movements per week as primary outcome, as recommended by the Food and Drug Administration (FDA) [47,48,49,50,51]. Overall, a significant improvement in bowel function has been reported, but the quality of evidence was deemed low, due to “indirectness, inconsistency, and imprecision across several outcomes” [17]. Therefore, AGA issued a conditional recommendation for methylnaltrexone in OIC, although emphasizing the advantage of the subcutaneous formulation in certain clinical situations. Naloxegol is supported by two RCTs [52,53], one open-label extension study [54], and one post hoc analysis of RCT [55]. Naloxegol proved to be more effective than placebo in all studies and to significantly reduce the median time to first post-dose spontaneous bowel movement. Naloxegol is also strongly recommended by the AGA for patients with laxative-refractory OIC, but the quality of evidence was rated moderate due to imprecision of the data [17].

5. *When prescribing a PAMORA:*
  - *Possible pharmacological interactions reported in the drug label should be carefully considered;*
  - *It is advisable to suspend or reduce concomitant laxatives at least for the first few days of PAMORA therapy, to avoid the risk of diarrhoea; laxatives can be reintroduced later based on individual needs;*
  - *The patient should be informed that the PAMORA must be taken regularly (and not as needed) and must not be self-suspended, unless adverse effects occur, for which he/she should consult the doctor.*

The class of PAMORA has some pharmacodynamic interactions that are of clinical interest. It is important to consider the influence of CYP450 enzymes and P-glycoprotein (P-gp) induction and inhibition on the plasma concentration of each PAMORA. The product labels of all PAMORAs warn of opioid withdrawal risk alone or when co-administered with opioid antagonists. Concomitant use of mixed partial agonists (e.g., pentazocine), partial agonist/antagonist opioid analgesics (e.g., buprenorphine) or combinations of opioid agonists combined with an antagonist (e.g., morphine/naltrexone) in patients taking any full agonist opioid (e.g., oxycodone) may reduce the analgesic effect or precipitate withdrawal symptoms [39]. Naloxegol and naldemedine have interactions with strong CYP3A4 inhibitors and inducers and strong P-gp inhibitors, which should be carefully checked in the Summary of Product Characteristics [56,57]. Methylnaltrexone bromide is minimally metabolized by CYP isozymes and does not affect the pharmacokinetics of medicinal products metabolized by cytochrome P450 (CYP) isozymes [58].

In some cases, introducing PAMORA in concomitance with laxatives may initially cause diarrhoea, which can be bothersome and worrisome for the patient and induce him/her to discontinue the PAMORA. For this reason, the expert panel recommends, upon prescribing PAMORA, to suspend the laxative and to re-evaluate at the subsequent follow-up the opportunity to reintroduce it or not and at which dosage on a case-by-case basis. Additionally, the panel recommends clearly explaining the characteristics of PAMORAs to patients and instructing them not to self-discontinue the PAMORA or take it on an as-needed-basis.

6. *Efficacy and tolerability of the PAMORA should be monitored regularly by the Bowel Function Index or a similar tool, and the OIC treatment adjusted accordingly.*

The course of constipation and any undesirable effects arising during treatment with PAMORAs must be closely monitored, at least every 2 weeks (after the first prescription of PAMORAs it is advisable to carry out a first check already after one week). PAMORAs are generally well tolerated. The most reported adverse effects are abdominal pain, diarrhoea, and nausea of mild to moderate severity, that generally resolve without discontinuation of the PAMORA treatment [55,56,57]. Diarrhoea was observed more frequently in cancer pain [43]. To evaluate bowel function adequately and discriminate changes in OIC, a standardized assessment tool, such as the BFI [38,39], is needed.

7. *After 4 weeks of treatment, if the efficacy of the PAMORA is deemed inadequate, the following options may be considered:*
  - *Discontinuation of the PAMORA;*
  - *Combination of the PAMORA with additional anti-constipation drugs;*
  - *Change of opioid type (and/or route of administration);*
  - *Refer the patient to a specialist at a dedicated referral centre.*

There is a lack of a clear definition of a time limit after which a PAMORA should be considered ineffective. Some evidence in the literature shows that a certain percentage of non-responders can be identified after 4 weeks of treatment with a PAMORA [59]. The panel agrees upon the fact that 4 weeks may be an adequate period to assess the effectiveness of PAMORAs in clinical practice. There is no evidence yet whether it is useful to change the PAMORA or add a second one after the first has not given an adequate response. Several other options can be considered in case the effectiveness of a PAMORA is deemed inadequate, as listed above. Adding laxatives, if not yet done, is probably the most obvious option, carefully modulating the dosage as needed. However, it is worth emphasizing that changing the opioid type or route of administration can represent a way of overcoming ineffective OIC therapy. Opioid switching may significantly improve the balance between analgesia and adverse effects of opioids. A switch to an alternative opioid has been reported to induce symptomatic relief in patients with inadequate analgesia or intolerable opioid-related adverse effects. However, data are based on open studies, small case series, and clinical experience [60,61,62]. If OIC treatment fails despite appropriate adjustments of therapy, the patients should be referred to a specialist for further investigations, ideally in a dedicated referral gastroenterology or internal medicine centre, as recommended by the ECHO OIC simplified pathway [21].

## 4. Conclusions

OIC is an extremely frequent side effect of chronic opioid therapy and typically does not improve upon opioid continuation. Despite its impact on patients’ QoL, direct/indirect costs, and the several existing guidelines, OIC is largely underdiagnosed and undertreated. The ECHO OIC European Committee recently proposed a simplified pathway specifically designed for primary care practitioners, with the aim of anticipating the recognition and improving the management of OIC [21,23]. This expert opinion was elaborated by a multidisciplinary group of 12 Italian healthcare professionals—including pain, cancer, GI specialists, internists, general practitioners, and a professional nurse—as a further step of the ECHO OIC project aimed at refining the European path taking into account the current Italian clinical practice. The starting and fundamental point of the path was identified in spreading knowledge of the OIC problem as much as possible to the health community in order to obtain not only an early treatment of the condition but also to promote its prevention. This is why it is crucial to also involve primary care professionals, especially with regard to chronic noncancer pain. Consistently with the European pathway, but going into some more useful detail for clinical practice, the expert panel defined a diagnostic–therapeutic pathway in seven main steps for the timely and appropriate management of OIC, which is suitable for both primary and secondary care.

## Figures and Tables

**Table 1:** The Rome IV diagnostic criteria for OIC [36].

**1**	New, or worsening, symptoms of constipation when initiating, changing, or increasing opioid therapy, that must include two or more of the following: Straining during more than ¼ (25%) of defecations; Lumpy or hard stools (Bristol Stool Form Scale 1–2) more than ¼ (25%) of defecations; Sensation of incomplete evacuation more than ¼ (25%) of defecations; Sensation of anorectal obstruction/blockage more than ¼ (25%) of defecations; Manual manoeuvres to facilitate more than ¼ (25%) of defecations (e.g., digital evacuation, support of the pelvic floor); Fewer than three spontaneous bowel movements per week.
**2**	Loose stools are rarely present without the use of laxatives.
